# The nonlinear energy model and stress–strain model of sandstone

**DOI:** 10.1038/s41598-023-35145-0

**Published:** 2023-05-25

**Authors:** Zhiming Zheng, Yu Yang, Cheng Pan

**Affiliations:** 1 School of Civil Engineering, Liaoning Technical University, Fuxin, 123000 China; 2 School of Civil Engineering, Liaoning University of Technology, Jinzhou, 123000 China

**Keywords:** Civil engineering, Mechanical engineering

## Abstract

The relationship between strain and elastic energy is simplified by introducing a stress state parameter based on the generalised Hooke’s law. It is assumed that the micro-element strengths satisfy the Weibull distribution and a new model for the non-linear evolution of energy is developed by introducing the concept of rock micro-element strengths. A sensitivity analysis of the model parameters is carried out on this basis. The results show that the model agrees well with the experimental data. The model is close to the deformation and damage laws of the rock and is able to reflect the relationship between the elastic energy and strain of the rock. By comparing with other model curves, the model of this paper is more suitable for the experimental curve. They show that the improved model could better describe the stress–strain relationship of rock. Finally, according to the analysis of the influence of the distribution parameter on the variation pattern of the elastic energy of the rock, the magnitude of the distribution parameter can directly reflect the peak energy of the rock.

## Introduction

The deformation and failure process of rock is the process of energy release, transformation, and dissipation, which leads to the imbalance of the original stable state of rock^1^. In practical engineering, the construction of an underground powerhouse, tunnel excavation, coal mining, and other engineering construction will destroy the original equilibrium state of rock^2,3^. It will cause the instability and failure of rock. During the construction of these projects, the energy inside the rock is constantly evolving. The mining and excavation of human activities is the process of external energy input into the rock. The construction measures such as reinforcement and support of underground engineering can be understood as the transformation and transfer of rock energy^4^. The deformation and failure of rock are dissipated or released in various forms of energy, such as heat energy, kinetic energy, and radiation energy. It can be seen that exploring the deformation and failure process of rock from the perspective of energy is closer to the engineering practice and the essence of rock failure. Therefore, revealing the law of energy change in the process of rock failure can provide important theoretical support for the construction of water conservancy projects^5^. With the increasing number of high-buried underground projects, the mining and excavation of deep rock have become the norm. In the complex stress state of rock material, the failure criterion of rock is complex^6^. Its damage constitutive model becomes very complex and changeable. Considering that energy drive is the essence of rock failure, the study of the constitutive model of rock materials from the perspective of energy can reduce the complexity of the damage constitutive model^7^. It can also fully show the deformation and failure process of rock materials. The establishment of an energy constitutive model of rock can deepen the understanding of the essence of the rock material failure process. The research results may be applied to a wider range of materials^8^.

The deformation process of the rock mass is accompanied by a change in energy, and energy is the fundamental internal cause of the ultimate damage to the driving material. The elastic energy stored in the rock during the loading process is sufficiently released to cause damage. During the process of sandstone failure under different confining pressures, the storage process of elastic energy has regular characteristics^9,10^. The rock damage process has been studied from the energy point of view. The ultimate goal is to discover the energy evolution mechanism of rock damage and find the rock damage prediction method based on the energy principle^11^. Through the energy accumulation and evolution characteristics of the sandstone failure process under different stress paths, the energy nonlinear evolution model of the rock failure process is established, and the energy prediction criterion of rock failure is given^12^.

Given the accumulation and consumption of energy in the process of rock deformation and destruction, the law of energy evolution can reflect the entire process of rock microstructure defects or structural degradation. Therefore, the analysis of energy evolution is beneficial to the study of rock deformation and failure. Zhang et al.^13^ studied the distribution of energy dissipation and release of rock under different impact loading rates and found that the dissipative energy of rock failure increases with the increase in loading rate. Meng et al.^14^ confirmed the rates of stored, elastic, and dissipated energies under different loading rates, presented an effective approach for the equivalent energy surface, and revealed the energy evolution of rock deformation and failure. Xiao et al.^15^ evaluated the advantages and disadvantages of different damage variables. Qiu et al.^16^ presented an incremental method and quantitatively analyzed the damage behavior of Jinping marble before peak stress. Based on uniaxial compression tests at different strain rates, Wang et al.^17^ found that the energy evolution of the karst limestone failure process has obvious stage characteristics, and the strain energy ratio is S-shaped. Li et al.^18^ redefined brittleness as the comprehensive ability to dissipate a small amount of energy in the pre-peak stage and maintain complete failure in the post-peak stage. It can accurately characterize the stress–strain curve and failure behavior of rock under different constraint levels. Zhang et al.^19^ conducted quasi-static compression tests on marble under different stress paths. It is found that the energy storage limit of rock samples under triaxial loading is higher than that under uniaxial compression or triaxial unloading. Based on the interaction mechanism of energy accumulation and energy dissipation, a nonlinear energy evolution model of rock is established.

The above research only analyzes the evolution law of rock energy and establishes the corresponding simple energy model. However, few energy models can describe the law of energy evolution. At the same time, fewer models can describe the stress–strain relationship using the energy model. In the current study, the conventional triaxial compression test of sandstone taken from Fuxin Hengda Coal Mine was carried out to analyze the stress–strain relationship of sandstone under different confining pressures and the mechanism of energy evolution and deformation failure. Then, an energy nonlinear evolution model was established by the energy release rate, the equivalent strain hypothesis principle, and the generalized Hooke’s law to predict the variation in the elastic energy with the change in strain during the loading process. Finally, the influence of distribution parameters on the change law of rock elastic energy is analyzed.

## Sandstone triaxial compression test study

### Test equipment and sample introduction

**Figure 1:**
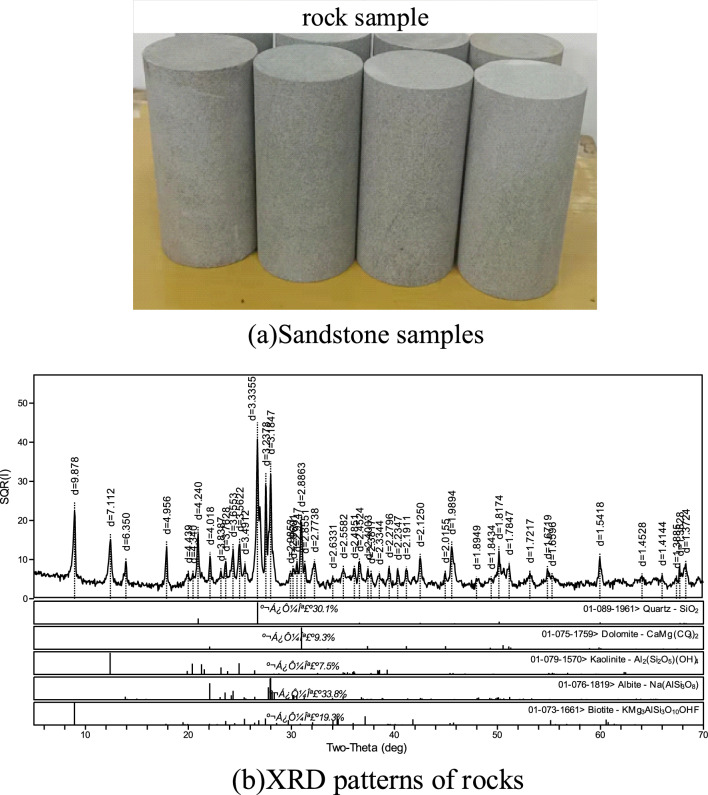
Sandstone samples.

The selected rock blocks are derived from the same section to reduce the differences in the samples and ensure the comparability of the tests. Figure 1a shows the sandstone samples. Post-processing test samples are screened by the above-mentioned accuracy requirements and examined visually. Samples with defects in appearance and evident differences are removed. The rock samples used for the test are all from Hengda Coal Mine. The depth of the selected samples is 800 ~ 850 m. The appearance of the rock sample is dark gray, the structure is relatively uniform, and the texture is relatively hard. There are no visible micro-cracks and bedding. To reduce the difference between the samples and ensure the comparability of the test, the selected rock blocks are all taken from the same section. The water content of the rock is 0.171%. The natural water absorption was 2.349%. The density is 2.355 g/cm^3^. Rock samples with similar wave velocities are selected as test pieces for the triaxial tests using the acoustic wave detection system of the MTS815.02 rock test machine. The grayish-white is primarily composed of quartz, albite, dolomite, biotite, feldspar, and kaolinite. The samples are processed in a standard cylinder with a diameter of 50 mm and a height of 100 mm. The XRD patterns of the rocks are shown in Fig. 1b.

**Figure 2:**
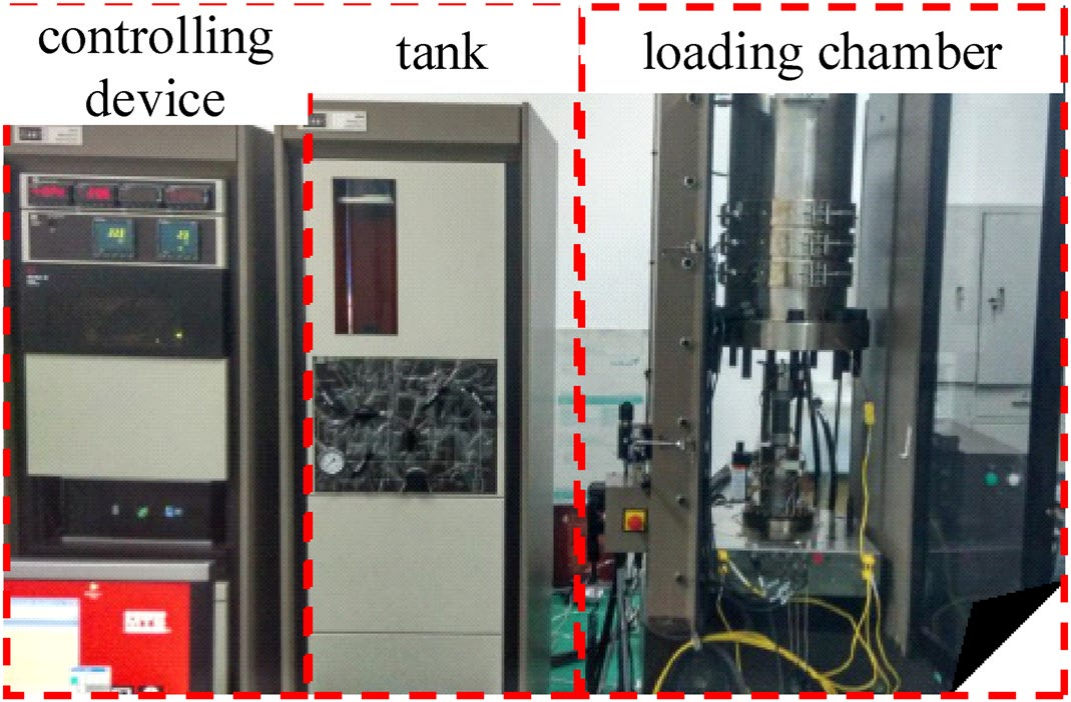
MTS815.02 Rock mechanics test system.

The main equipment used in this study is a multifunctional electro-hydraulic servo-controlled rigid testing machine that is specially developed for analyzing rock and concrete (MTS Corporation, USA). The test system consisted of a loading part, a test part, and a control part. The device has three independent closed-loop servo control functions each for controlling the axial, confining, and water pressures. The equipment test data are objective and reliable. The double-mean axial extensometer and circumferential extensometer achieve high test accuracy, and multiple servo control methods are available. The major technical parameters of the test machine are as follows: a stiffness of 7.0 × 109 N/m, a maximum axial pressure of 1600 KN, a maximum confining pressure of 70 MPa, and a maximum pore water pressure of 70 MPa. Figure 2 shows the test equipment.

### Test plan

The confining pressures of 0, 10, 20, 30 and 40 MPa are used in the test. To ensure the correctness of the stress–strain curve of the conventional triaxial compression test under each confining pressure, three specimens are selected for testing under each confining pressure condition. Due to the high in-situ stress of the rock, the maximum horizontal in-situ stress is 45 MPa and the vertical in-situ stress is 25 MPa. Therefore, this area belongs to a high-ground stress environment. According to this value, the confining pressure value of this paper is formulated.

Table 1 shows the specific test loading scheme.

**Table 1:** Test plan.

Stress path	Sample no.	Confining pressure/MPa
Uniaxial compression	A11	0
Conventional triaxial compression	B11, B21, B31	10
Conventional triaxial compression	B12, B22, B32	20
Conventional triaxial compression	B13, B23, B33	30
Conventional triaxial compression	B14, B24, B34	40

The peak strength of three samples needs to be tested. When the difference between the three peak strengths does not exceed 15%, three peak strengths are taken as the final peak strength of the rock sample. When the difference between any two of the three peak strengths exceeds 15%, the mechanical property test needs to be restarted until the above conditions are met^20^. When satisfying the above conditions, the data corresponding to the intermediate peak intensity is selected as the research object of this paper.

### Test steps

The test steps of the mechanical properties of rock under different confining pressures are as follows.

1. Both ends of the sandstone sample are evenly smeared with vaseline to ensure that the measured test data are not affected by the end effect.
2. Sandstone samples need to be placed in the center of the test bench. It is necessary to cover the thermoplastic film on the outside of the sandstone and ensure that the thermoplastic film is close to the sample. Axial and radial displacement sensors need to be installed. The sample and tester are placed in the center of the pressure chamber and the pressure hood is slowly lowered and the hydraulic oil is filled into the pressure chamber.
3. The loading rate of confining pressure and axial pressure is 0.5 mm/min.
4. The confining pressure needs to be loaded to a predetermined value. It is necessary to keep the confining pressure value constant during the loading process.
5. The axial compression needs to be continuously loaded until the residual stage.

The test data were saved and exported, and the stress–strain curves under different confining pressure conditions are plotted.

The test data are automatically collected by the testing machine and converted into corresponding strain and stress output to the data acquisition system. Table 2 shows the results of the triaxial compression test. *σ*_1c_ is the peak stress. *ε*_1c_ is the peak strain. *σ*_1r_ is the residual stress. *ε*_1r_ is the residual strain.

**Table 2:** Triaxial compression test results.

Confining pressure/MPa	(Peak stress)*σ*_1c_/MPa	(Peak strain)*ε*_1c_	(Residual stress)*σ*_1r_/MPa	(Residual strain)*ε*_1r_
0	76.07	0.0032	2.78	0.0035
10	98.23	0.0035	39.41	0.0069
20	112.95	0.0037	63.54	0.0083
30	116.66	0.0041	79.35	0.0103
40	127.56	0.0043	92.30	0.0113

**Figure 3:**
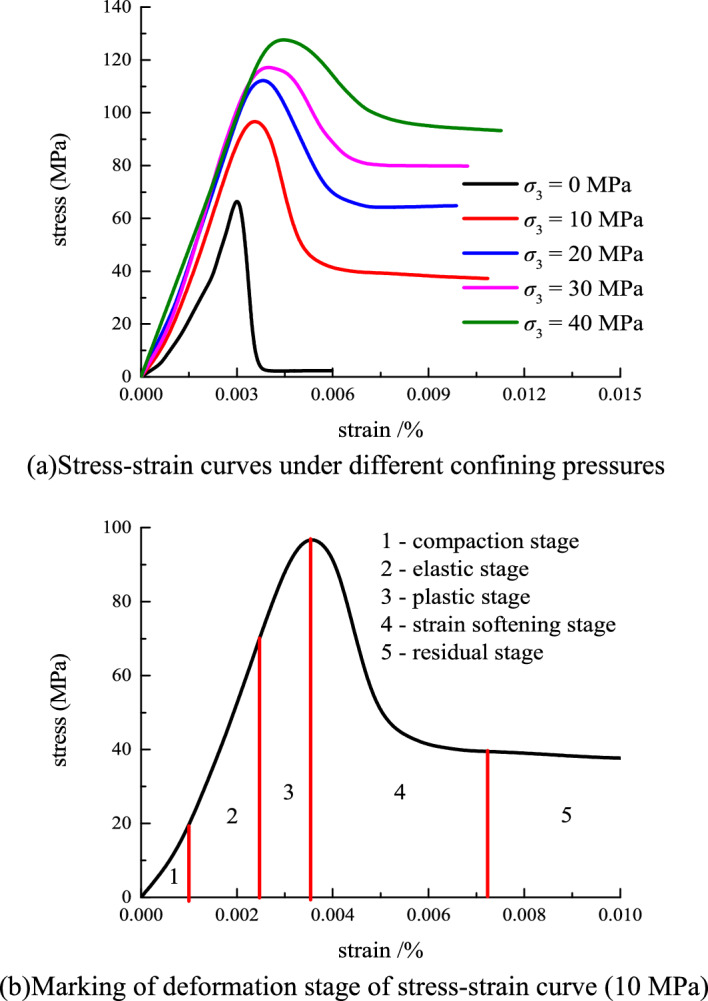
Axial stress–strain curves.

Figure 3 shows the axial stress–strain curve.

Figure 3 shows that the stress–strain relationship curves under different confining pressures are the same, and both show typical sandstone brittle characteristics. In the compaction stage, the original pores inside the rock gradually close under the external load. As a result, the strain doesn’t change considerably. The curves are coincident and showed no evident deviation. In the elastic phase, the strain is restrained due to the increase in confining pressure. As the axial strain increases with the increase in the deviatoric stress, the curve shows an evident deviation. In the plastic phase, as the confining pressure increases, the degree of deviation of the curve and the stress corresponding to the peak point of the rock increases. In the post-peak softening stage, the stress decreases rapidly and gradually stabilizes as the strain gradually increased. Compared with those in uniaxial compression, the peak strength and strain, and residual strength of conventional triaxial loading damage are considerably improved. In the triaxial test, an evident confining pressure effect is observed. As the confining pressure increases, the peak intensity increases evidently. A high confining pressure indicates a high peak intensity. The residual strength also has a strong confining pressure effect. As the confining pressure increases, the residual strength increases.

## Analysis of rock failure energy evolution characteristics

### Principle of energy calculation

By the laws of thermodynamics and under the external load, the external force is calculated as the input energy of the rock^21^.

1 $$ U = U_{e} + U_{d} $$

where *U*_e_ is the elastic energy, *U*_d_ is the dissipative energy, and *U* is the total energy.

During rock loading, external loads will cause external heat loss when working on the rock. For ease of calculation, this study ignores energy loss in this part. This study considers that the external load produces work on the rock equal to the energy absorbed by the rock from the outside. The energy that the rock absorbs from the outside is the sum of elastic energy and dissipated energy. In the rock triaxial compression test, the energy absorbed by the rock includes the sum of the works done by the axial and radial pressures. The work done by the axial compression is the same as that of the axial compression in the uniaxial state, but the radial coercive work on the rock is due to the radial dilatancy damage. Therefore, the radial work is negative. The energy absorbed by the rock from the outside is calculated as follows:

2 $$ U = U_{1} + U_{3} $$

where *U*_1_ is the axial strain energy and *U*_3_ is the radial strain energy.

The axial and radial strain energies can be expressed as

3 $$ U_{1} = \int {\sigma_{1} d\varepsilon_{1} = \sum\limits_{i = 0}^{n} \frac{1}{2} } \left( {\varepsilon_{{1\left( {i + 1} \right)}} - \varepsilon_{1i} } \right)\left( {\sigma_{{1\left( {i + 1} \right)}} + \sigma_{1i} } \right), $$

4 $$ U_{3} = 2\int {\sigma_{3} d\varepsilon_{3} = \sum\limits_{i = 0}^{n} {\left( {\varepsilon_{{3\left( {i + 1} \right)}} - \varepsilon_{3i} } \right)\left( {\sigma_{{3\left( {i + 1} \right)}} + \sigma_{3i} } \right)} } , $$

where *σ*_1i_, *σ*_3i_ and *ε*_1i_, *ε*_3i_ are the axial radial stress and strain of the point on the full stress–strain curve of the corresponding triaxial compression test, respectively.

The triaxial compression elastic energy calculation equation is

5 $$ \begin{gathered} U_{e} = \frac{1}{2E}\left[ {\sigma_{1}^{2} + \sigma_{2}^{2} + \sigma_{3}^{2} - 2\nu \left( {\sigma_{1} \sigma_{2} + \sigma_{1} \sigma_{3} + \sigma_{2} \sigma_{3} } \right)} \right]\mathop = \limits^{{\sigma_{2} = \sigma_{3} }} \hfill \\ \frac{1}{2E}\left[ {\sigma_{1}^{2} + 2\sigma_{3}^{2} - 2\nu \left( {2\sigma_{1} \sigma_{3} + \sigma_{1} \sigma_{3} + \sigma_{3}^{2} } \right)} \right], \hfill \\ \end{gathered} $$

where *E* is the initial elastic modulus, υ is the Poisson’s ratio.

### Analysis of sandstone loading and failure energy evolution

1. Total energy determination method.

Through the software Origin, the axial stress–strain and circumferential data stress–strain are integrated by area respectively, and the new data after integration are obtained. The total energy in the rock loading process is obtained by adding the integrated data.

(2) Determination method of element elastic energy.

Firstly, the elastic modulus and Poisson’s ratio of rock under different confining pressures are determined. The axial stress–strain, circumferential stress–strain data, elastic modulus, and Poisson’s ratio under different confining pressures are substituted into Eq. (5). The elastic energy data of rock during loading can be determined.

(3) Dissipative energy determination method.

The total energy minus the elastic energy is the dissipated energy of the rock.

**Figure 4:**
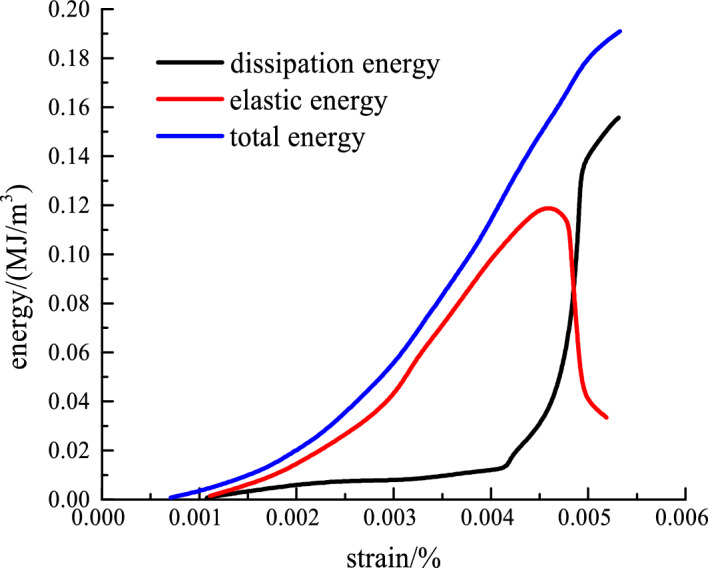
Uniaxial energy evolution curve.

Figure 4 shows the uniaxial energy test curve by the above-mentioned energy calculation equation.

In the initial loading stage, the elastic energy density curve of the rock coincides with the total energy density curve. At this time, the dissipation energy curve is the sum of the x-coordinate axis weight. After the elastic strain stage, although the elastic energy curve continues to increase, the dissipation energy curve also begins to show an increasing trend. At this time, the growth rate of elastic energy is less than that of dissipative energy. After the peak point, as the strain increases, the elastic energy begins to decrease and the dissipation energy begins to increase. When the elastic energy of rock exceeds the limited energy storage, it will begin to release at the peak stress, which will lead to the deformation and failure of sandstone. In the process of energy release, the tiny cracks inside the rock will gradually form a macroscopic crack and gradually develop a fracture surface.

**Figure 5:**
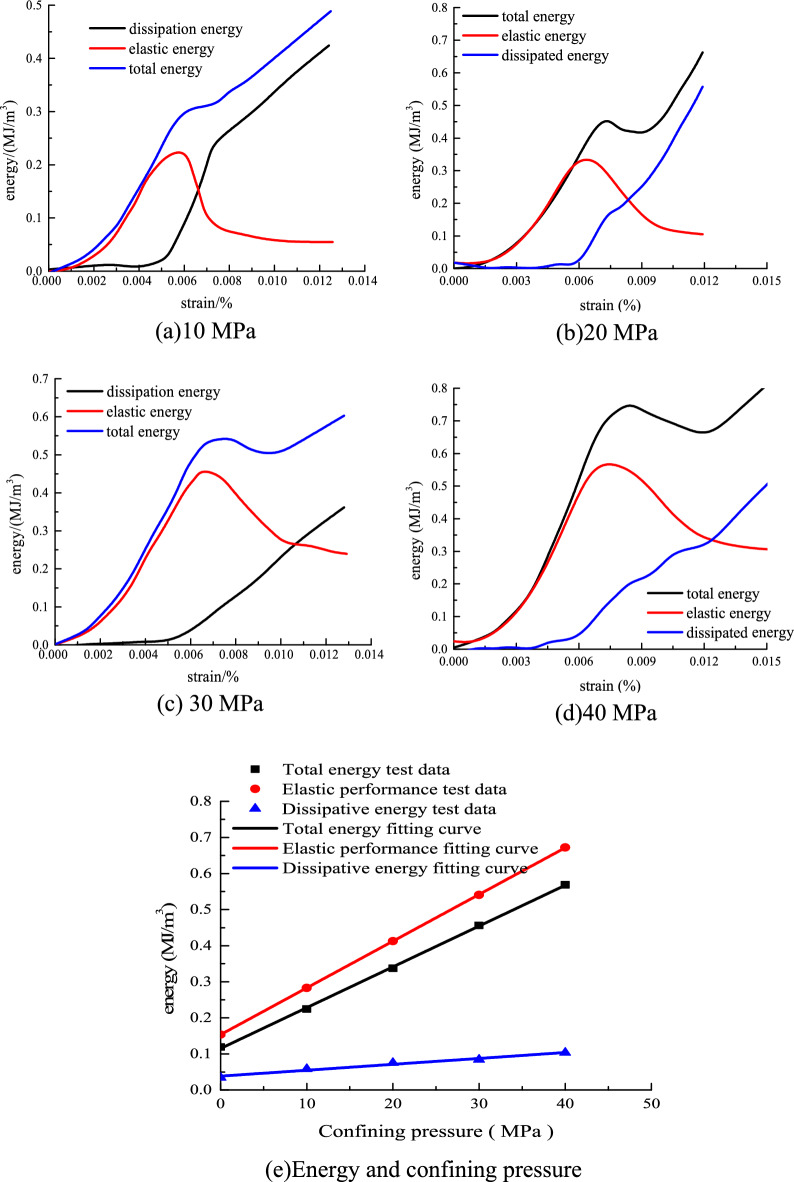
Triaxial compression energy evolution curve.

Figure 5 shows the triaxial compression energy test curve by the aforementioned energy calculation equation.

Figure 5 shows that, as the confining pressure increases, the external input of energy into the rock as the elastic energy also increases. The elastic energy growth trend is consistent with the rock stress–strain curve change law. With the increase of strain, the variation of elastic energy also shows a trend of increasing first and then decreasing and then regional stability. The elastic energy-strain curve also has obvious peak points. The total energy and elastic energy curves coincide before elastic deformation. After that, the total energy and elastic energy still increased, but the growth rate slowed down. The elastic energy begins to release near the peak stress. It led to the deformation and failure of sandstone. At this time, the dissipation energy begins to increase sharply, but the elastic energy is still greater than the dissipation energy. The rock strain enters the yield plastic stage, and a large number of new cracks and cracks are generated inside the sandstone. The energy consumed by rock deformation also rises sharply. Part of the cracks is connected to form a new fracture surface. When the stress reaches the peak, the strain energy reaches the maximum (exceeding the limited energy storage capacity of sandstone). The elastic energy begins to release and causes the deformation and instability of sandstone, but the change rate of total energy shows a downward trend. At the peak point, all of the stored energy is released, thereby causing rock damage and the start of the post-peak residual phase. When the confining pressure is 10 MPa, the total energy of the rock tends to change after the peak point. At this time, the total energy curve shows a law of first decreasing and then increasing. The total energy of confining pressure 30 MPa has a clear trend of decreasing first and then increasing after the peak. The total energy absorbed by the rock sample includes the positive work done by the axial force and the negative work done by the radial force. The compressive strain is positive and the circumferential strain is negative. The larger the confining pressure value is, the more negative work the radial confining pressure does, and the total energy curve shows a downward trend. In turn, the total energy curve will show a short downward trend after the peak. This is because the radial strain value of rock is greater than the axial strain value when the confining pressure is larger in the initial post-peak period. However, both axial and radial strain growth rates decrease with the duration of load. And the negative work done by the radial confining pressure is less than the positive work done by the axial stress. This causes the total energy curve to gradually increase again. When the surrounding rock is 10 MPa, the total energy curve does not show an obvious downward trend in the post-peak stage. It could be that the negative work done by the confining pressure is less than the positive work done by the axial force^22^.

Figure 5e shows that, as the confining pressure increases, the elastic and total energies of the rock show a linear increasing trend at the peak point. The energy in conventional triaxial compression is considerably greater than the energy in uniaxial compression. Therefore, the increase in confining pressure gradually increases the energy storage limit and the total energy of the input rock system and effectively improves the bearing capacity of the rock.

The variation law of the energy evolution curve is analyzed according to the deformation stage of the stress–strain curve. (1) Pore compaction stage. The total energy input to the rock by the external testing machine is mainly converted into elastic energy. At this stage, the curves of elastic energy and total energy coincide. With the increase of strain, the curves show a trend of increasing concave. The dissipated energy almost coincides with the strain axis. But there is still some capacity that is released to the outside world in various forms. This is because, in the stage of compaction and deformation of rocks, part of the energy needs to be used for the motion of void compaction and particle friction. (2) Elastic deformation stage. At this stage, the curves of total energy and elastic energy still coincide. However, the curve also deviates from the increased axial strain. At this time, the dissipated energy of the rock starts to deviate from the strain axis and has an obvious trend of increasing. The proportion of dissipated energy to total energy has increased. This is because, at this stage, the rock began to produce new cracks and other internal defects under the action of external loads. The generation of these defects and their subsequent development and expansion require energy consumption. (3) Plastic yield stage. At this stage, cracks and other defects in the rock are more completely developed. At this time, the dissipated energy of the rock increases sharply. The elastic energy also increases with the increase of axial strain value. But the growth rate of elastic energy starts to slow down. It can be considered that the main cause of rock failure is the massive release of elastic energy. (4) Post-peak strain-softening stage. At this stage, the elastic energy of the rock decreases with the increased axial strain value. The relationship between the total energy and the dissipated energy of the rock and the strain in the post-peak deformation stage satisfies the linear change relationship. At this time, the number and development speed of cracks in the rock is also faster. The mutual motion between the microscopic particles is more frequent, and the sliding and dislocation of the fracture surface are more intense. Therefore, the energy consumed by rock deformation in the post-peak stage is also more. (5) Residual deformation stage. At this stage, the elastic energy of the rock remains unchanged with the increasing axial strain value. The total energy and dissipated energy of rock still increase linearly with the increase of strain.

The corresponding energy values at the peak points of the selected elastic energy curve are shown in Table 3.

**Table 3:** Peak point energy value.

Confining pressure/MPa	*U*_e_/(MJ/m^3^)	*U*/(MJ/m^3^)	*U*_d_/(MJ/m^3^)
0	0.1193	0.1537	0.0344
10	0.2245	0.2830	0.0585
20	0.3374	0.4128	0.0754
30	0.4564	0.5408	0.0844
40	0.5689	0.6723	0.1034

## Establishment of statistical damage constitutive model for rock damage

In the study of rock strength and overall failure criterion based on the principle of energy dissipation and release, Xie et al.^23^ found that the maximum energy release rate occurs in the direction of minimum compressive stress. When the maximum energy release rate *G*_3_ reaches the critical value *Gc*, the strain energy stored in the unit will be released along this direction first. When the elastic strain energy of the unit reaches the surface energy required for the overall failure of the rock mass unit, the elastic strain energy of the unit will be completely released. The unit’s body is suddenly destroyed. When the two energies are equal, the unit body undergoes static overall failure. When the elastic strain energy of the element is greater than the surface energy, the unit body undergoes dynamic overall failure, and the energy difference constitutes the kinetic energy of the split unit body.

6 $$ G_{3} = K_{3} \left( {\sigma_{1} - \sigma_{3} } \right)U_{e} , $$

where *K*_3_ is the material constant.

When the maximum energy release rate *G*_3_ reaches the critical value *G*_c_, the strain energy stored in the unit will be first released in this direction, that is, *G*_3_ = *G*_c._

7 $$ \left\{ \begin{gathered} U_{e}^{^{\prime}} = \sigma_{c}^{3} /2E \hfill \\ G_{c} = \left( {K_{3} \sigma_{c}^{3} } \right)/2E \hfill \\ \end{gathered} \right., $$

where *σ*_c_ is the uniaxial compressive strength of the rock, *W*^’^_e_ is the elastic energy in the uniaxial state, and *E* is the elastic modulus.

Under the condition of *G*_3_ = *G*_c_, substituting Eq. (7) into Eq. (6) yields

8 $$ \left( {\sigma_{1} - \sigma_{3} } \right)U_{e} = \sigma_{c}^{3} /2E. $$

The generalized Hooke’s law and the equivalent strain principle posit that the rock satisfies the following conditions under normal triaxial compression.

9 $$ \left\{ {\begin{array}{*{20}c} {\varepsilon_{1} = \frac{1}{{E\left( {1 - D} \right)}}\left[ {\sigma_{1} - 2\nu \sigma_{3} } \right]} \\ {\varepsilon_{3} = \frac{1}{{E\left( {1 - D} \right)}}\left[ {\sigma_{3} - \nu \left( {\sigma_{3} + \sigma_{1} } \right)} \right]} \\ \end{array} } \right., $$

where *μ* is the Poisson’s ratio, *ε*_1_ is the axial strain, *ε*_3_ is the radial strain, and *D* is the damage variable.

The axial strain *ε*_1_ and the radial strain *ε*_3_ are subtracted to obtain.

10 $$ \varepsilon_{1} - \varepsilon_{3} = \frac{1 + \nu }{{\left( {1 - D} \right)E}}\left( {\sigma_{1} - \sigma_{3} } \right). $$

Equation (10) is substituted into Eq. (8) to obtain the relationship between the elastic energy and the strain.

11 $$ U_{e} = \frac{{\left( {1 + \nu } \right)\sigma_{c}^{3} }}{{2E\left( {\varepsilon_{1} - \varepsilon_{3} } \right)(1 - D)E}}. $$

During the rock loading process, the Mohr–Coulomb failure criterion not only effectively reflects the deformation and failure of geomaterials but also effectively describes the strength characteristics of the rock. This criterion has been widely used in the field of geotechnical engineering and is expressed as

12 $$ F = \sigma_{1} - \alpha \sigma_{3} { - }\beta = {0,} $$

where *α* and *β* are the test parameters, and *F* is the yield function.

In general, the test parameters α and *β* satisfy the following conditions:

13 $$ \left\{ \begin{gathered} \alpha = \tan^{2} \left( {\pi /4 + \varphi /2} \right) \hfill \\ \beta = 2c\tan^{2} \left( {\pi /4 + \varphi /2} \right) \hfill \\ \end{gathered} \right., $$

where *c* is the cohesive force and *φ* is the internal friction angle.

When the radial strain *ε*_3_ is used in Eq. (11), the elastic energy and the axial strain cannot establish a good correspondence. Therefore, the relationship between axial and radial strain must be obtained to establish a nonlinear model of energy evolution.

In the uniaxial stress state, the radial-axial strain relationship of the ideal elastomer is

14 $$ \varepsilon_{3} = - \nu \varepsilon_{1} . $$

Under the triaxial stress state, the radial-axial strain relationship of the ideal elastomer is

15 $$ \frac{{\varepsilon_{3} }}{{\varepsilon_{1} }} = \frac{{\sigma_{3} \left( {1 - \nu } \right) - \mu \sigma_{1} }}{{\sigma_{1} - 2\nu \sigma_{3} }}, $$

The radial-axial strain relationship of the ideal elastomer under unidirectional and triaxial stress states can be assumed as

16 $$ \frac{{\varepsilon_{3} }}{{\varepsilon_{1} }} = - \lambda , $$

where *λ* is the coefficient related to the stress state.

In the unidirectional stress state, the coefficient *λ* related to the stress state satisfies the condition

17 $$ \lambda = \nu . $$

In the 3D stress state, the coefficient λ related to the stress state satisfies the condition

18 $$ \lambda = - \frac{{\sigma_{3} \left( {1 - \nu } \right) - \nu \sigma_{1} }}{{\sigma_{1} - 2\nu \sigma_{3} }}. $$

The relationship between axial and radial strains is substituted into the elastic energy model.

19 $$ U_{e} = \frac{{\left( {1 + \nu } \right)\sigma_{c}^{3} }}{{2E\varepsilon_{1} \left( {1 + \lambda } \right)(1 - D)E}}. $$

The damage variable is an internal variable that describes the stress–strain change and the deterioration of mechanical properties inside the rock. The Weibull distribution in statistical damage mechanics describes not only the progressive distribution of material damage well but also the damage evolution law and failure process curve of rock. Therefore, the damage characteristics of the rock are assumed to satisfy the Weibull distribution.

According to Kachanov’s definition of damage, the damage to a material can generally be represented by the number of internal damage units and the ratio of the total number of materials^24^.

20 $$ D = N_{f} /N, $$

where *D* is the damage variable, *N* is the total number of rock elements, and *N*_*f*_ is the number of rock element failures.

The strength of the rock micro-element is assumed to satisfy the Weibull distribution function when damage occurs.

The probability density function *f*(*F*) can be expressed as

21 $$ f\left( F \right) = \frac{n}{{F_{0} }}\left( {\frac{F}{{F_{0} }}} \right)^{n - 1} \exp \left( { - \left( {\frac{F}{{F_{0} }}} \right)^{n} } \right), $$

where *n* and *F*_0_ are the distribution parameters.

Microscopically, when the rock is subjected to the external load to the yield strength, the number of micro-injections in the rock can reach *N*_*f*_^25^.

22 $$ N_{f} = \int_{0}^{F} {Nf\left( x \right)} dx. $$

The damage evolution equation of rock is obtained from Eqs. (20), (21) and (22).

23 $$ D = 1 - \exp \left( { - \left( {\frac{F}{{F_{0} }}} \right)^{n} } \right). $$

The damage model is substituted into the elastic energy model, and the constitutive equation of the energy nonlinear evolution of the rock is obtained.

24a $$ U_{e} = \frac{{\left( {1 + \nu } \right)\sigma_{c}^{3} }}{{2E^{2} \varepsilon_{1} \left( {1 + \lambda } \right)\exp \left( { - \left( {\frac{F}{{F_{0} }}} \right)^{n} } \right)}}. $$

Substituting Eq. (24a) into Eq. (8), Eq. (24b) can be obtained.

24b $$ \sigma_{1} = \frac{{\left( {1 + \lambda } \right)E\exp \left( { - \left( {\frac{F}{{F_{0} }}} \right)^{n} } \right)}}{{\left( {1 + \nu } \right)}}\varepsilon_{1} + \sigma_{3} . $$

## Determination of parameters of the nonlinear evolutionary constitutive model of rock energy

*E* and* v* can be calculated by the stress–strain curve under different confining pressure conditions. The distribution parameters *m* and *F*_0_ are generally determined by the relationship of the special points of the stress–strain curve.

### Determination of distribution parameters

**Figure 6:**
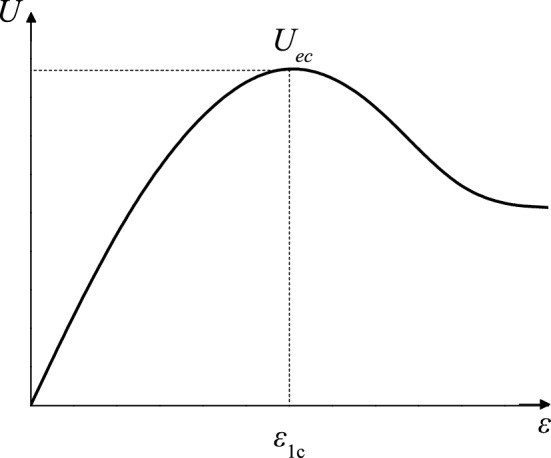
Diagram of rock energy evolution curve.

The schematic of the rock stress–strain curve in Fig. 6 shows that the following geometric relationships exist when the rock is loaded and deformed to the peak point^26^.

Condition (1) *U*_e_ = *U*_ec_ is satisfied when *ε*_1_ = *ε*_1c_.

Condition (2) ∂*U*_e_/∂*ε* = 0 is satisfied when *ε*_1_ = *ε*_1c_.

By combining conditions (1) and (24a), (b), Eq. (25) can be obtained.

25 $$ U_{ec} = \frac{{\left( {1 + \nu } \right)\sigma_{c}^{3} }}{{2E^{2} \varepsilon_{1c} \left( {1 + \lambda_{c} } \right)\exp \left( { - \left( {\frac{F}{{F_{0} }}} \right)^{n} } \right)}} $$

By combining conditions (2) and (24a), (b), Eq. (26) can be obtained.

26 $$ \frac{{\partial U_{e} }}{{\partial \varepsilon_{1} }}\left| {_{{\varepsilon_{1} = \varepsilon_{1c} ,U_{e} = U_{ec} }} } \right. = 0. $$

Equation (12) is the yield function of macroscopic rock. By substituting the damage variable, it can be transformed into the yield function of the rock element. The strength criterion of rock micro-elements can be expressed as

27 $$ F^{*} = \frac{{\left( {\sigma_{1} - \alpha \sigma_{3} } \right)E\varepsilon_{1} }}{{\sigma_{1} - 2\nu \sigma_{3} }} - \beta . $$

The first derivative of the elastic energy to the axial strain is

28 $$ \frac{{\partial U_{e} }}{{\partial \varepsilon_{1} }} = \frac{{\left( {1 + \nu } \right)\sigma_{c}^{3} }}{{2E^{2} \left( {1 + \lambda } \right)}}\frac{1}{{\varepsilon_{1}^{2} }}\left\{ {\exp \left( {\left( {\frac{F}{{F_{0} }}} \right)^{n} } \right)\frac{n}{F}\left( {\frac{F}{{F_{0} }}} \right)^{n} \frac{\partial F}{{\partial \varepsilon_{1} }} - \exp \left( {\left( {\frac{F}{{F_{0} }}} \right)^{n} } \right)} \right\}. $$

The first derivative of the yield function to axial strain is

29 $$ \frac{\partial F}{{\partial \varepsilon_{1} }} = \frac{{\left( {\sigma_{1} - \alpha \sigma_{3} } \right)E}}{{\sigma_{1} - 2\mu \sigma_{3} }} + \frac{{\left( {\alpha - 2\nu } \right)\sigma_{3} E\varepsilon_{1} }}{{\left( {\sigma_{1} - 2\nu \sigma_{3} } \right)^{2} }}\frac{{\partial \sigma_{1} }}{{\partial \varepsilon_{1} }}. $$

By substituting Eqs. (28) and (29) into Eq. (26), the simultaneous equation gives the distribution parameter *n* as

30 $$ n = \frac{{BF_{1c} }}{{\left( {1 - D_{1c} } \right)\ln \left( {1 - D_{1c} } \right)}}, $$

where *F*_1c_ is the rock strength value at the peak point and *D*_1c_ is the damage variable value at the peak point.

*B* is

31 $$ B = \frac{{\left( {\sigma_{1c} - 2\mu \sigma_{3} } \right)^{2} }}{{\left( {\sigma_{1c} - \alpha \sigma_{3} } \right)E^{2} \varepsilon_{1c}^{2} }}. $$

The distribution parameter *m* is substituted into Eq. (23) to obtain the distribution parameter *F*_0_.

32 $$ F_{0} = F_{1c} \left[ { - \ln \left( {1 - D_{1c} } \right)} \right]^{\frac{1}{n}} . $$

The intensity value of the rock at the peak point, the value of the damage variable, and the coefficient *λ* can be expressed as

33 $$ F_{1c} = E\varepsilon_{1c} + \left( {2\nu - \alpha } \right)\sigma_{3} { - }\beta , $$

34 $$ D_{1c} = 1 - \frac{{\left( {1 + \nu } \right)\sigma_{c}^{3} }}{{2E^{2} \varepsilon_{1c} \left( {1 + \lambda_{c} } \right)U_{ec} }}, $$

35 $$ \lambda_{c} = - \frac{{\sigma_{3} \left( {1 - \nu } \right) - \nu \sigma_{1c} }}{{\sigma_{1c} - 2\nu \sigma_{3} }}. $$

### Determination of physical parameters of rock mechanics

The elastic parameters of sandstone under different confining pressure conditions from the triaxial laboratory test data are shown in Table 4.

**Table 4:** Sandstone elastic parameters.

Confining pressure/MPa	*E*/GPa	*υ*
0	28.12	0.234
10	30.05	0.262
20	31.45	0.276
30	33.82	0.312
40	35.77	0.354

**Figure 7:**
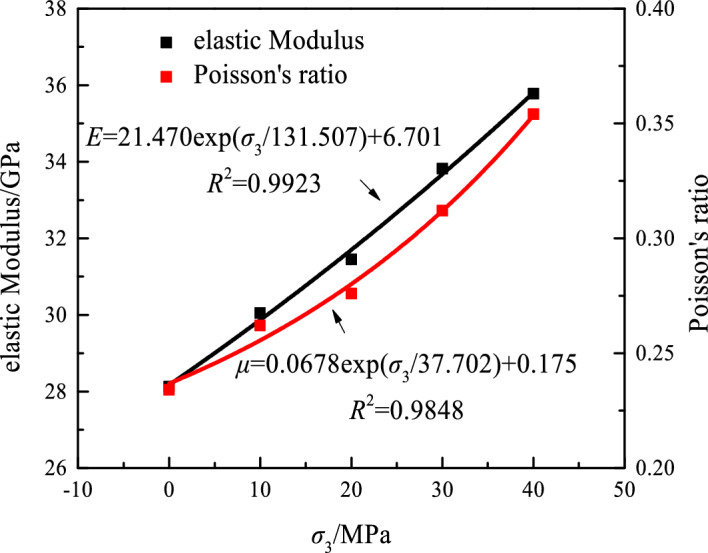
Relationship among confining pressure, Poisson’s ratio, and elastic modulus.

The variation in the elastic modulus and Poisson’s ratio of rock under different confining pressure conditions is plotted based on the data in Fig. 7.

Figure 7 shows that, as the confining pressure increases, the elastic modulus, and the Poisson’s ratio increase. As the confining pressure further increases, the rock resists deformation and can withstand loads, thereby making sandstone less prone to damage.

**Figure 8:**
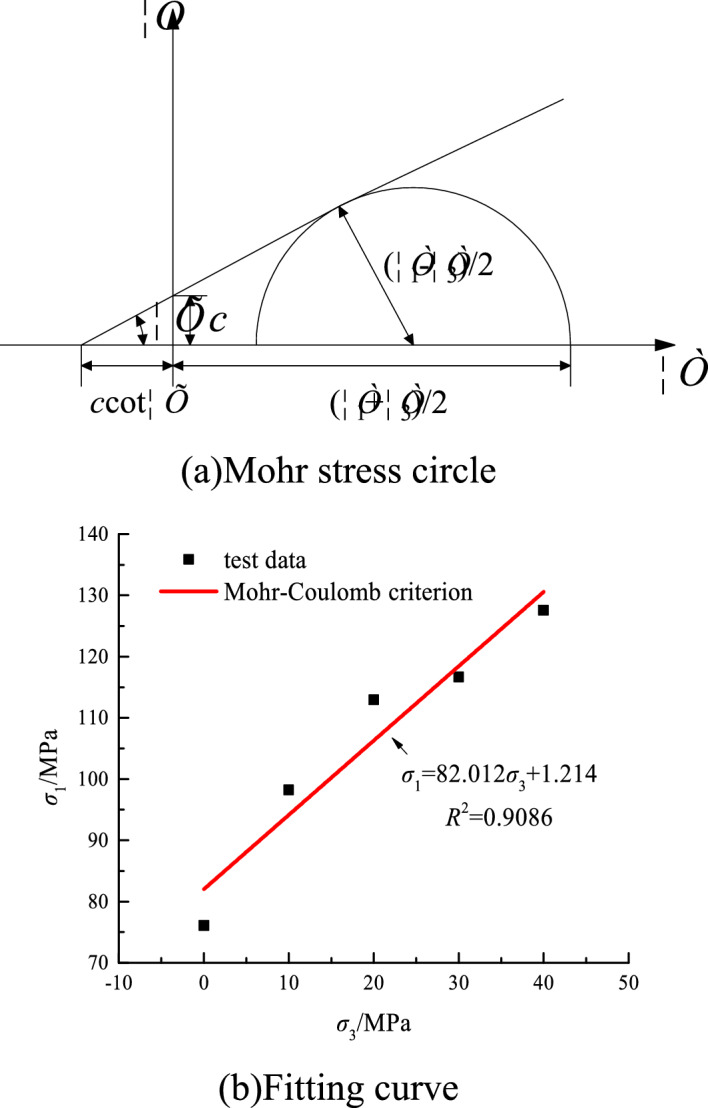
Fitting curve of the first and third principal stresses.

The cohesive force and internal friction angle of the rock are determined by the peak strength of the rock under different confining pressures. The first and third principal stress test data and the Mohr–Coulomb criterion fitting curve are shown in Fig. 8.

According to Fig. 8a, the axial pressure and confining pressure satisfy the following relationship.

36 $$ \sigma_{1} = \frac{2c\cos \varphi }{{1 - \sin \varphi }} + \sigma_{3} \frac{1 + \sin \varphi}{{1 - \sin \varphi }}, $$

where *c* is cohesion, *φ* is the internal friction angle.

Equation (36) can be transformed as follows.

37 $$ \sigma_{1} = A + B\sigma_{3} , $$

where *A* and *B* are substitution parameters.

*A* and *B* can be expressed as

38 $$ A = \frac{2c\cos \varphi }{{1 - \sin \varphi }},\,B = \frac{1 + \sin \varphi }{{1 - \sin \varphi }}. $$

From Eq. (38), the cohesion and internal friction angle can be inversely calculated as

39 $$ \varphi = \sin^{ - 1} \frac{B - 1}{{B + 1}},\,c = A\frac{1 - \sin \varphi }{{2\cos \varphi }}. $$

The cohesion *c* = 14.43 MPa and the internal friction angle *φ* = 27.48°. The Mohr–Coulomb criterion has a good fitting degree with the experimental data. This condition indicates that this failure criterion can be used to describe the rock deformation and failure characteristics.

### Determination of rock damage model parameters

The values of each parameter in the statistical damage constitutive model are shown in Table 5.

**Table 5:** Parameter value.

Confining pressure/MPa	0	10	20	30	40
*n*	0.513	0.468	0.437	0.382	0.350
*F*_0_	28.702	24.854	21.542	18.426	19.236
*R*^2^	0.951	0.964	0.937	0.968	0.983
Reduced Chi-Sqr	0.132	0.290	0.023	0.014	0.026

The calculated values of the above-mentioned parameters are only the distribution parameter values under a specific confining pressure and cannot fully represent the relationship between the distribution parameters and the confining pressure in all cases.

**Figure 9:**
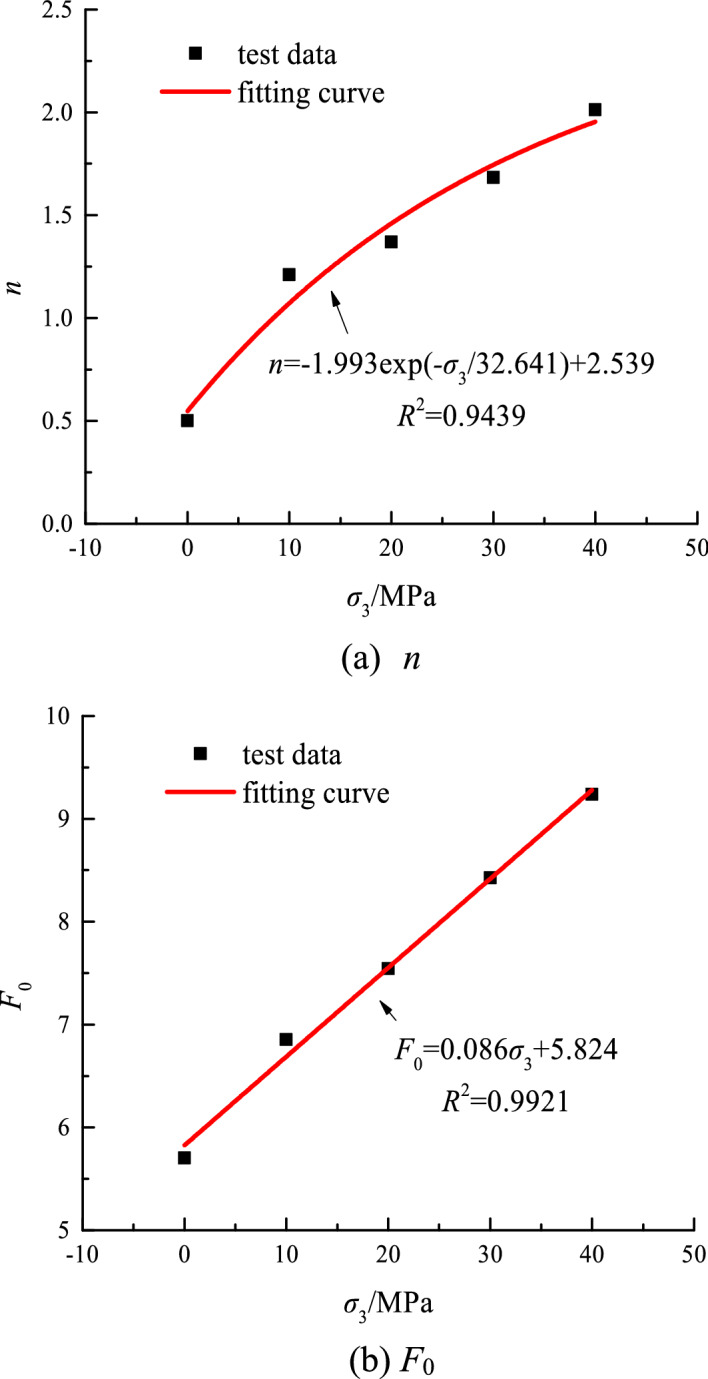
Relationship of distribution parameters and confining pressure.

Therefore, the parameters are appropriately corrected by the distribution parameters under different confining pressures. The correction curve is shown in Fig. 9. The correction equation is presented as follows.

40 $$ n{ = } - {1}{\text{.993exp(}} - \sigma_{{3}} {/32}{\text{.641) + 2}}{.539,} $$

41 $$ F_{0} = 5.824 + 0.086\sigma_{3} . $$

The final elastic energy model equation can be obtained by substituting formula (40) and formula (41) into formula (24a).

42 $$ U_{ec} = \frac{{\left( {1 + \nu } \right)\sigma_{c}^{3} }}{{2E^{2} \varepsilon_{1c} \left( {1 + \lambda_{c} } \right)\exp \left( { - \left( {\frac{F}{{5.824 + 0.086\sigma_{3} }}} \right)^{{{ - 1}{\text{.993exp( - }}\sigma_{{3}} {/32}{\text{.641) + 2}}{.539}}} } \right)}}. $$

### Model validation and comparison

**Figure 10:**
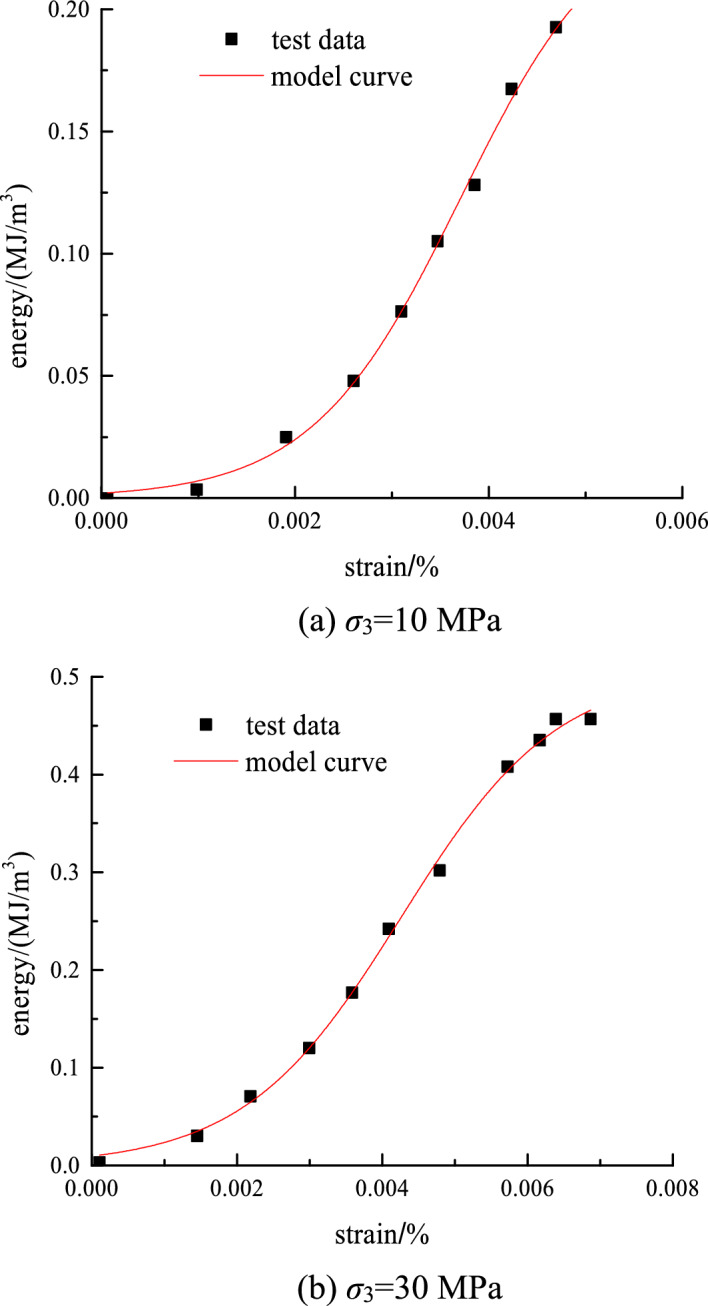
Comparison of test data and model curve.

The distribution parameter correction equation is substituted into the elastic energy model to obtain the rock nonlinear energy evolution model. By substituting different confining pressure values and elastic modulus and Poisson’s ratio values of rock into the energy model equation, the energy model curve of rock under different surrounding rock effects can be obtained. The energy evolution and test curves of the sandstone model under different confining pressures of 10 and 30 MPa are plotted in Fig. 10.

The model curve has a high degree of fitting with the experimental data, and its correlation coefficient is large at above 0.98. Therefore, the model is close to the actual damage and evolution law of surrounding rock, and the established model can effectively reflect the elastic energy–strain relationship of rock. Most of the parameters in the proposed model can be determined through experiments, and the introduced distribution parameters have evident physical meaning.

The distribution parameter values are substituted into the damage model.

43 $$ D = 1 - \exp \left( { - \left( {\frac{F}{{5.824 + 0.086\sigma_{3} }}} \right)^{{ - {1}{\text{.993exp(}} - \sigma_{{3}} {/32}{\text{.641) + 2}}{.539}}} } \right) $$

**Figure 11:**
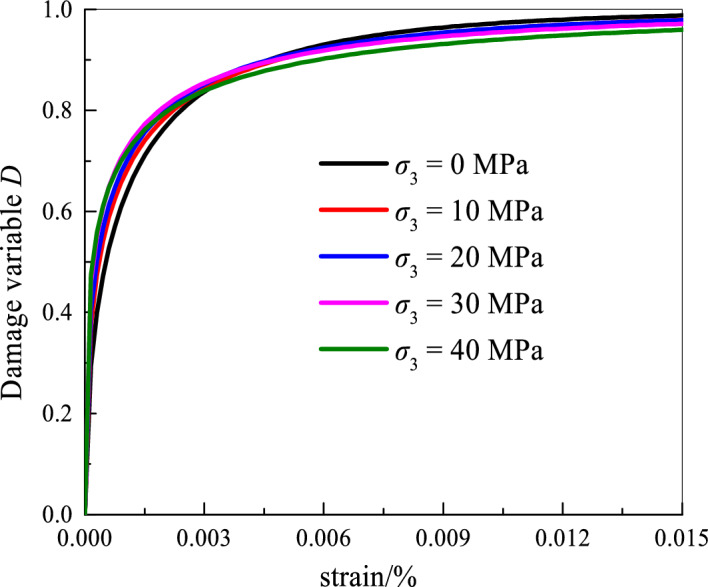
Damage evolution.

The damage evolution law of surrounding rock under different confining pressures is shown in Fig. 11.

Figure 11 shows that, under the same confining pressure, the damage variable increases with the increase in the axial strain. The micro-cracks also gradually expand. When the axial strain is approximately 0.0015%, the damage value reaches nearly 0.7. The damage occurs in the plastic phase after the elastic phase. That is, after the stress–strain curve transitions from the elastic phase to the plastic phase, the damage develops sharply. In the plastic damage stage, a small confining pressure indicates a small damage value. At the initial moment of loading, the internal pores are compacted to decrease the damage changes. However, as the load increases, the internal pores are compacted and the rock produces cracks. The damage trend increases with the cumulative effect of microcracks and cracks. At the same time, the microcracks converge in weak areas, and the damage rate is further increased and approaches 1. This process is consistent with the damaged evolution of the actual rock. This consistency indicates the rationality and correctness of the damage evolution model.

**Figure 12:**
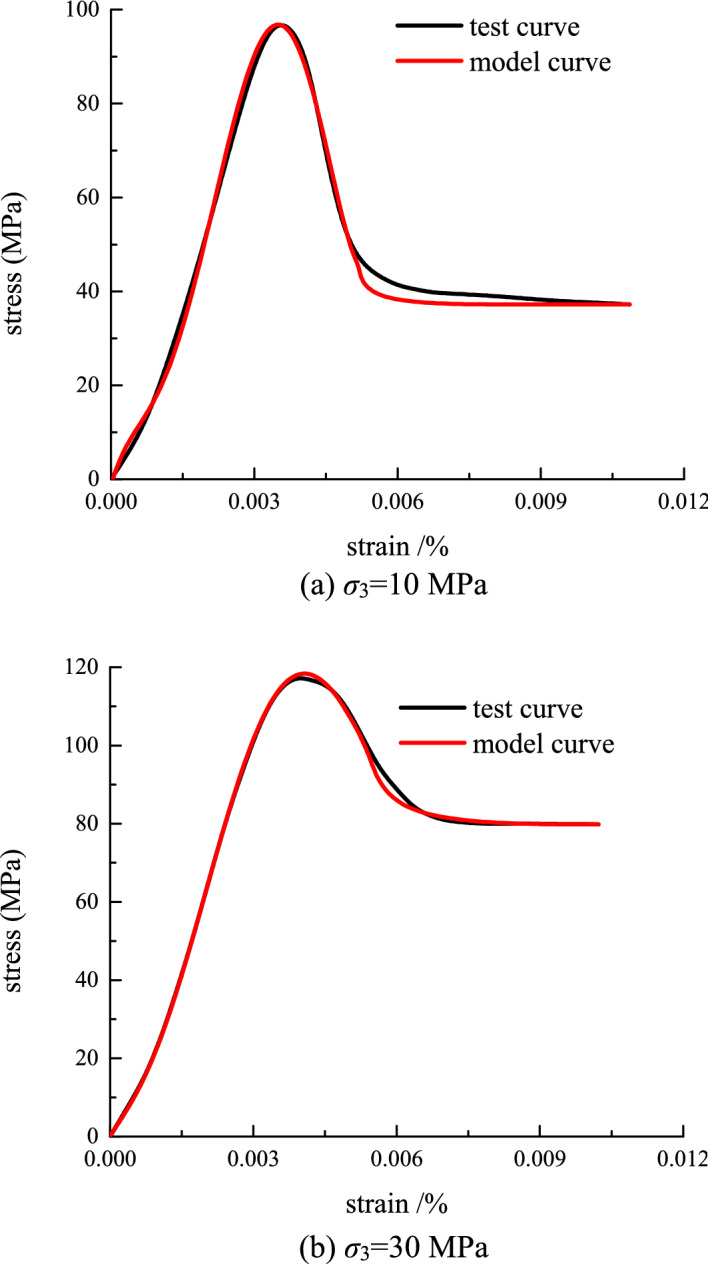
The comparison between the model curve of the stress–strain relationship and the test curve.

Substituting the obtained distribution parameters into Eq. (25), the comparison between the model curve of the stress–strain relationship and the test curve can be obtained(as shown in Fig. 12).

It can be seen from Fig. 12 that the model established in this paper is in better agreement with the experimental curve than the experimental curve. Especially in the post-peak strain softening stage, the model curve in this paper is more in line with the experimental curve. Therefore, the damage constitutive model established in this paper can better reflect the stress–strain variation law of rock under different confining pressures. It provides a theoretical basis for anchoring support in practical engineering.

### Influence of distribution parameters on elastic energy

**Figure 13:**
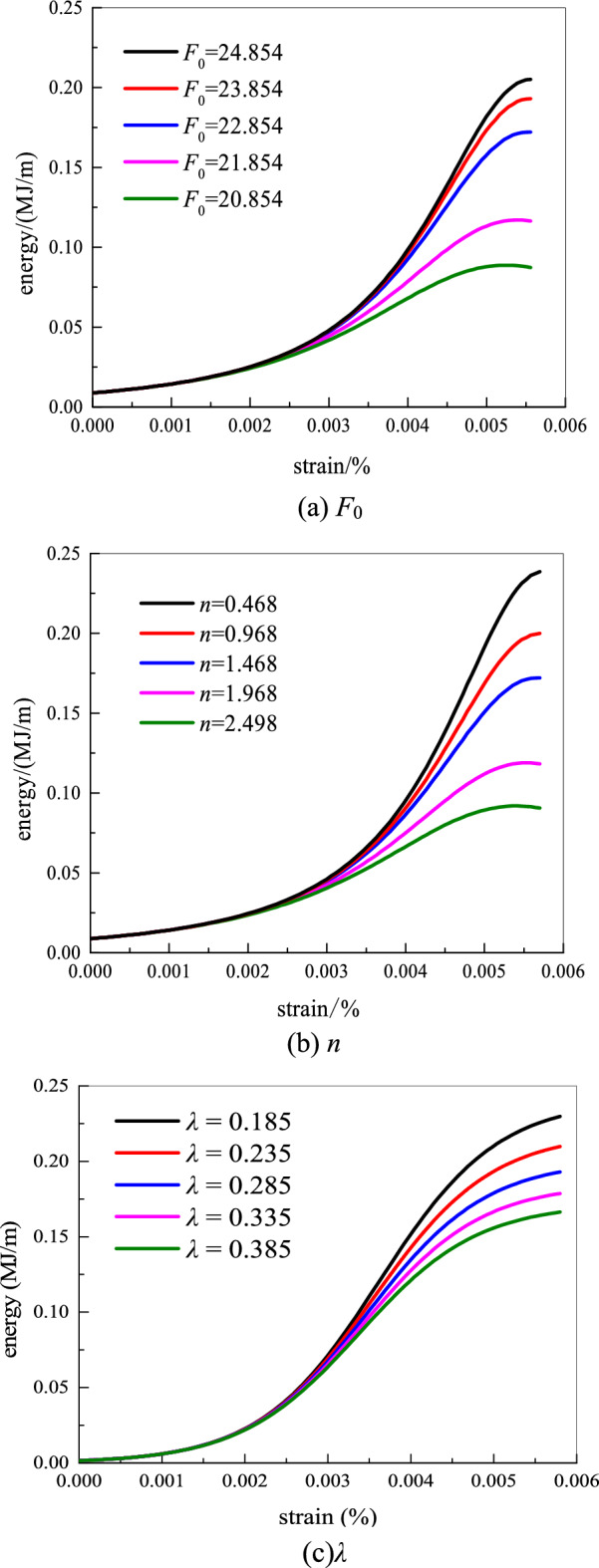
Effect of distribution parameters on the elastic performance.

In the verification of the above-mentioned sandstone nonlinear energy model, the influence of the distribution parameters on energy evolution is not analyzed. Thus, the importance of each distribution parameter in the energy model is explored under the confining pressure of 10 MPa. The relationship between the distribution parameters m and *F*_0_ on energy and strain is discussed. The law of influence is shown in Fig. 13.

When *n* is fixed, the elastic energy of sandstone increases with the increase in *F*_0_. The reason is that the damage variable *D* gradually decreases as *F*_0_ increases. The ability of sandstone to resist deformation and damage from external loads is increased, which slows down the expansion of sandstone microfractures. The sandstone has low energy loss in the compression test, and a large amount of energy is available to convert the work with an external load. According to the law of conservation of energy, the amount of elastic energy stored in the rock is large. When *F*_0_ is fixed, the elastic energy decreases with the increase in *n*. The internal deformation and damage of sandstone are gradually intensified, and the development of cracks on the microscopic surface is complete. A large amount of energy is consumed during rock loading, thereby restoring a small amount of elastic energy inside the rock. Therefore, the distribution parameters *F*_0_ and *n* effectively reflect the evolution law of rock energy. The values of these parameters can be used to determine rock energy and damage. Under the same strain, as the parameter *λ* increases, the energy decreases, and the energy curve becomes more and more gentle. This also shows that the increase of the parameter *λ* reduces the energy storage inside the rock. When the strain value is 0.058% and the distribution parameter *F*_0_ is reduced by 4 values, the elastic energy is reduced by 59.71%. When the strain value is 0.058% and the distribution parameter *n* increases by 2 values, the elastic energy is reduced by 67.79%. When the strain value is 0.058% and the parameter *λ* increases by 0.2, the elastic energy is reduced by 27.95%.

## Discussion

**Figure 14:**
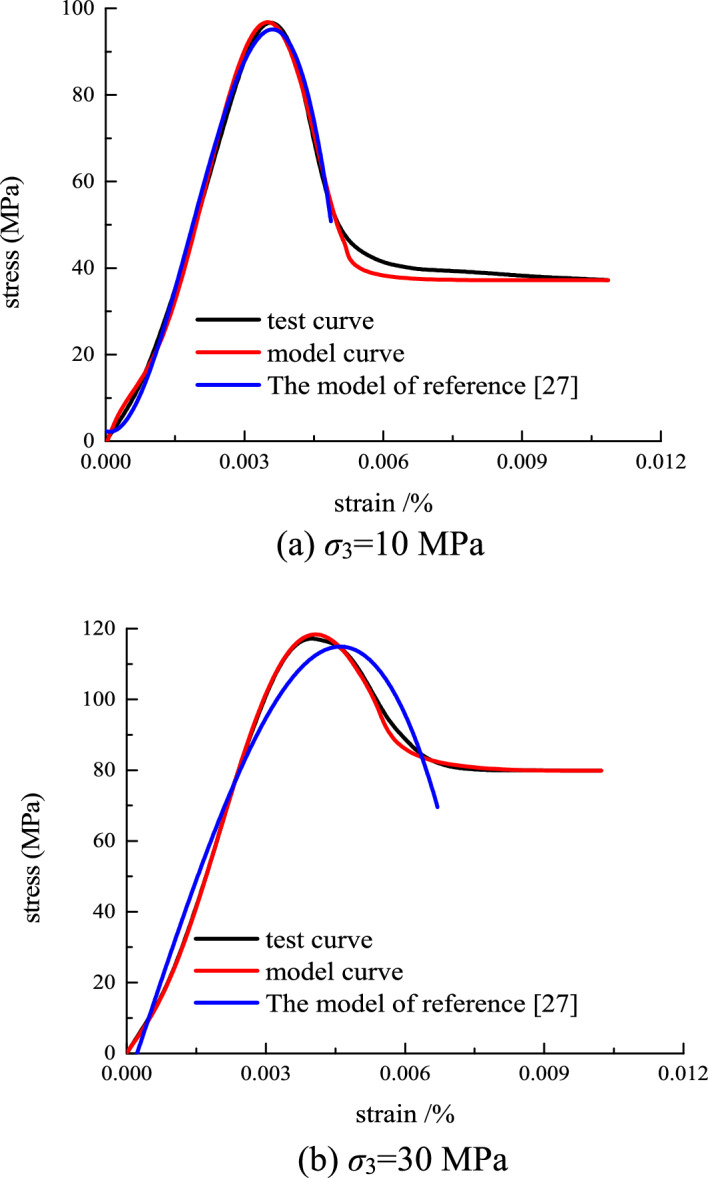
Comparison of the literature model and model curve in this paper.

To further verify the correctness and superiority of the model established in this paper, the model in Reference^27^ is used to compare the model with the test curve. The comparison curve is shown in Fig. 14.

The model in reference^27^ can describe the stress–strain curve of rock well. However, it cannot describe the residual deformation stage of the stress–strain curve. At the same time, the coincidence of the stress–strain curve of the rock in the post-peak softening stage is lower than that of the proposed model. By comparing with other model curves, the model of this paper is more suitable for the experimental curve. They show that the improved model could better describe the stress–strain relationship of rock.

## Conclusions

1. The energy nonlinear evolution model of rock failure is consistent with the experimental data. The energy fitting curve is consistent with the test curve. The correlation coefficient is above 0.95. It shows that the establishment of various nonlinear energy models can better reflect the energy evolution law in the process of rock failure. However, the energy model cannot describe the post-peak energy stage and will continue to be studied in subsequent studies.
2. The model parameters can better reflect the evolution of rock energy. It provides a method to judge the changing trend of rock energy by model parameter values.
3. The constitutive model that can describe the stress–strain curve is obtained by differentiating the energy model. The model curve is in good agreement with the experimental curve. The model can also well describe the stress–strain curve in the post-peak stage.
4. When the strain value is 0.058% and the distribution parameter F0 is reduced by 4 values, the elastic energy is reduced by 59.71%. When the strain value is 0.058% and the distribution parameter n increases by 2 values, the elastic energy is reduced by 67.79%. When the strain value is 0.058% and the parameter λ increases by 0.2, the elastic energy is reduced by 27.95%.

## Data Availability

The datasets used and/or analyzed during the current study are available from the corresponding author upon reasonable request.
